# Antipsychotic-Induced Movement Disorders in Long-Stay Psychiatric Patients and 45 Tag SNPs in 7 Candidate Genes: A Prospective Study

**DOI:** 10.1371/journal.pone.0050970

**Published:** 2012-12-04

**Authors:** P. Roberto Bakker, Asmar F. Y. Al Hadithy, Najaf Amin, Cornelia M. van Duijn, Jim van Os, Peter N. van Harten

**Affiliations:** 1 Psychiatric Centre GGZ Centraal, Amersfoort, The Netherlands; 2 Hospital Pharmacy, Erasmus MC, Rotterdam, The Netherlands; 3 Department of Epidemiology, Erasmus MC, Rotterdam, The Netherlands; 4 Department of Psychiatry and Psychology, South Limburg Mental Health Research and Teaching Network, EURON, Maastricht University Medical Centre, Maastricht, The Netherlands; 5 Department of Psychosis Studies, Institute of Psychiatry, King’s Health Partners, King’s College London, London, United Kingdom; Oslo University Hospital, Norway

## Abstract

**Objective:**

Four types of antipsychotic-induced movement disorders: tardive dyskinesia (TD), parkinsonism, akathisia and tardive dystonia, subtypes of TD (orofacial and limb truncal dyskinesia), subtypes of parkinsonism (rest tremor, rigidity, and bradykinesia), as well as a principal-factor of the movement disorders and their subtypes, were examined for association with variation in 7 candidate genes (*GRIN2B*, *GRIN2A*, *HSPG2*, *DRD3*, *DRD4*, *HTR2C*, and *NQO1*).

**Methods:**

Naturalistic study of 168 white long-stay patients with chronic mental illness requiring long-term antipsychotic treatment, examined by the same rater at least two times over a 4-year period, with a mean follow-up time of 1.1 years, with validated scales for TD, parkinsonism, akathisia, and tardive dystonia. The authors genotyped 45 tag SNPs in 7 candidate genes, associated with movement disorders or schizophrenia in previous studies. Genotype and allele frequency comparisons were performed with multiple regression methods for continuous movement disorders.

**Results:**

Various tag SNPs reached nominal significance; TD with rs1345423, rs7192557, rs1650420, as well as rs11644461; orofacial dyskinesia with rs7192557, rs1650420, as well as rs4911871; limb truncal dyskinesia with rs1345423, rs7192557, rs1650420, as well as rs11866328; bradykinesia with rs2192970; akathisia with rs324035; and the principal-factor with rs10772715. After controlling for multiple testing, no significant results remained.

**Conclusions:**

The findings suggest that selected tag SNPs are not associated with a susceptibility to movement disorders. However, as the sample size was small and previous studies show inconsistent results, definite conclusions cannot be made. Replication is needed in larger study samples, preferably in longitudinal studies which take the fluctuating course of movement disorders and gene-environment interactions into account.

## Introduction

Soon after the introduction of antipsychotic medication in 1952, movement disorders emerged as a complication of treatment. To date, they remain a major concern in antipsychotic treatment. Of the different movement disorders, tardive dyskinesia (TD) is the most extensively investigated. TD and other movement disorders are associated with social stigmatization, physical disabilities and poorer quality of life. In addition, they play a role in non-compliance and, therefore, risk of psychotic relapse [1]–[3].

A central problem in the management of movement disorders is the lack of clear genetic and non-genetic risk factors that would allow for early identification and prevention. It would be helpful if movement disorders could be predicted from a minimal number of genetic susceptibility loci in candidate genes in combination with demographic, clinical or pharmacological data. In order to identify individuals at risk, pharmacogenetic studies of genetic factors that contribute to interpersonal differences in susceptibility for medication-related adverse effects are needed [4]. Family studies suggest an important genetic component to the risk for movement disorders [4]–[9]. A recent meta-analysis on the prevalence of dyskinesia and parkinsonism reported spontaneous dyskinesia and parkinsonism in antipsychotic naïve patients with schizophrenia, and a higher prevalence of dyskinesia and parkinsonism in healthy family members of patients with schizophrenia, compared to matched controls [10].

Antipsychotic-induced movement disorders [11], [12] can be classified as acute or tardive. Acute syndromes appear within hours/days or weeks after starting antipsychotics or increasing the dosage (or cessation of anticholinergics). Examples of these are parkinsonism and akathisia. Tardive syndromes develop after months or years of treatment with antipsychotics, examples being TD and tardive dystonia. Initially, the term ‘tardive’ (delayed) was introduced to emphasize the late-onset types of movement disorders occurring during antipsychotic use. Yet the definition of tardive disorders in the current study emphasizes their persistence, which is clinically more important than their late-onset [12], [13]. Given that combinations of acute and chronic movement disorders occur in patients undergoing long-term treatment with antipsychotics, prediction models should include both syndromes, i.e., the four major types of movement disorders (TD, parkinsonism, akathisia and tardive dystonia). It is noteworthy that movement disorders may fulfill the criteria for classifying a trait as a spectrum condition of a disorder, in this case schizophrenia: heritability, familial link, co-segregation, and biological and clinical plausibility [14]. Spectrum conditions refer to mild psychopathology of little clinical significance among relatives without the full disorder. The advantage for research of spectrum conditions in contrast to a full disorder is that they may have fewer risk factors and therefore a less complex chain of mechanisms (pathways) leading to their onset, which could make research easier to perform. (Pharmaco) genetic studies may help elucidate these common pathways in the development of both spectrum conditions and the full disorder. It is possible to hypothesize that specific subtypes of movement disorders are more suitable for genetic analysis than a general movement disorder syndrome, as subtypes may better reflect the underlying biological heterogeneity in complex syndromes.

The phenotypes under study were TD, parkinsonism, akathisia, and tardive dystonia, subtypes of TD (orofacial and limb truncal dyskinesia), subtypes of parkinsonism (rest tremor, rigidity, and bradykinesia), as well as a principal-factor of the movement disorders and their subtypes.

The 7 candidate genes were *GRIN2B*, *GRIN2A*, *HSPG2*, *DRD3*, *DRD4*, *HTR2C*, and *NQO1* (Text S1). The choice of these genes was hypothesis-driven, under the common disease/common variant (CDCV) hypothesis, which proposes that common diseases may be caused by common genetic variants [15]–[18].

The aim of the current study was to determine the association between movement disorders and variations in these 7 candidate genes.

The prospective design of the current study extends hitherto cross-sectional work in the pharmacogenetic field of antipsychotic-induced movement disorders. Indeed, prospective assessment of fluctuating (repeated) movement disorders measures the phenotype more specifically and that increases the validity of the associations between movement disorders and risk factors.

## Methods

### Ethics Statement

The protocol was approved by the standing Institutional Review Board, ‘Medisch-ethische Toetsingscommissie Instellingen Geestelijke Gezondheidszorg’ (Review Board for Human Research in Psychiatry), the Netherlands [protocol number 377].

Written informed consent was obtained from each patient, hence, consent obtained from the next of kin was not necessary and not recommended by the Review Board for Human Research in Psychiatry.

### Subjects

A 4-year prospective naturalistic study (July 2003– May 2007) was conducted with 209 patients with chronic mental illness in order to determine the genetic risk factors of the four major types of movement disorders (TD, parkinsonism, akathisia, and tardive dystonia), subtypes of TD and parkinsonism, as well as a principal-factor of the movement disorders and their subtypes. To this end, a cohort was drawn from a general psychiatric hospital (GGZ Centraal, Amersfoort, the Netherlands). Full details of the study design and movement disorders have been published previously [19] (Bakker and colleagues, submitted). The cohort was representative of the population of patients with the most severe chronic mental illness requiring long-stay care, given that the hospital serves an epidemiological catchment area, is the only institute providing this type of care in this area, and patients were selected from a comprehensive list of all inpatients.

Of the patients assessed at baseline (N = 207) 93.7% (n = 194) had at least one follow-up and 59.4% (n = 123) had two follow-up assessments. Loss to follow-up was due to patients who were difficult to trace after leaving hospital, died or refused assessment after inclusion.

### Assessment

Patients were examined by a trained psychiatrist (PRB), using a standard protocol, described by van Harten and colleagues [20]. In addition, subtypes of movement disorders were assessed using (i) the Abnormal Involuntary Movement Scale (AIMS) [21], [22] with items 1–4 for orofacial and items 5–7 for limb truncal dyskinesia, (ii) the Unified Parkinson Disease Rating Scale (UPDRS) [23] with item c3–c4 for ‘rest tremor’ (rest tremor, and action/postural tremor of hands); item c5 for rigidity; and items c1, c2, c6–c12, and c14 for bradykinesia. This approach has been described previously by 3 members of our research team (AAH, JvO and PNvH) [24]–[26].

As movement disorders likely share genetic liability, a genetic association between the combined movement disorders and candidate genes is also required. To determine the association between the combined movement disorders and variation in 7 candidate genes, a principal-factor of the four major types of movement disorders and subtypes of TD and parkinsonism was calculated with the FACTOR procedure in the STATA statistical program [27].

Based on the literature published between 1976 and July 2012, we selected 7 candidate genes (Tables 1 and S1, and Text S1) that are involved in the dopaminergic and serotonergic systems, and protection of neurotoxicity, and we included the gene coding for heparan sulfate proteoglycan 2, all which have been implicated in the development of movement disorders.

**Table 1:** Selected 45 tag SNPs for multilevel regression of continuous movement disorders.

Gene	Tag SNP	Chromosome Position	Alleles Public	ReferencesSNPs/Genes	TD^a^		PK^a^		AK^a^		TDt^a^		PF^a^	
			Major/Minor	Demonstratedassociations	*Beta*	*p-value*	*Beta*	*p-value*	*Beta*	*p-value*	*Beta*	*p-value*	*Beta*	*p-value*
**GRIN2B**		chr12∶13,714,410–14,133,022												
	rs1805481	chr12∶13,763,205–13,763,705	AC		−0.03	0.6062	0.05	0.3529	−0.04	0.5621	−0.02	0.2882	0.08	0.4914
	rs7313149	chr12∶13,828,037–13,828,537	TC		0.02	0.7816	0.03	0.5460	−0.01	0.8739	0.00	0.9931	0.02	0.8586
	rs2192970	chr12∶13,836,063–13,836,563	CT		−0.12	0.1007	−0.07	0.2263	0.04	0.6385	0.00	0.7915	−0.14	0.2406
	rs2300242	chr12∶13,840,047–13,840,547	TA		0.01	0.9097	0.05	0.3792	0.01	0.9256	−0.01	0.4171	0.12	0.2904
	rs10845838	chr12∶13,894,146–13,894,646	GA		0.06	0.4429	−0.01	0.8834	−0.11	0.1218	−0.02	0.1964	0.08	0.4910
	rs12300851	chr12∶13,968,155–13,968,655	TC		–	–	–	–	–	–	–	–	–	–
	rs220599	chr12∶13,975,048–13,975,548	GA		−0.02	0.7367	0.04	0.4773	−0.01	0.8670	−0.01	0.4562	0.07	0.5170
	rs10772715	chr12∶14,037,753–14,038,253	GA		−0.10	0.1086	−0.07	0.1479	−0.02	0.7624	−0.01	0.3444	−0.20	0.0362
	rs12827536	chr12∶14,095,907–14,096,407	CT		−0.04	0.4927	0.02	0.6875	−0.02	0.7378	−0.01	0.4348	−0.04	0.7222
**GRIN2A**		chr16∶9,847,265–10,276,263												
	rs11866328	chr16∶9,862,306–9,862,806	GT		0.12	0.0512	−0.02	0.7185	0.04	0.4868	0.02	0.2581	−0.02	0.8312
	rs11646587	chr16∶9,873,069–9,873,569	GA		−0.03	0.6401	−0.06	0.3363	0.02	0.7751	−0.02	0.2945	−0.11	0.3275
	rs7196095	chr16∶9,885,582–9,886,082	TC		0.02	0.7279	−0.02	0.7082	0.09	0.1678	−0.01	0.6353	−0.03	0.8048
	rs8049651	chr16∶9,943,416–9,943,916	CT		0.05	0.4769	0.02	0.7706	0.01	0.9313	−0.00	0.8313	0.08	0.4938
	rs9989388	chr16∶9,965,889–9,966,389	CT		−0.03	0.6765	0.08	0.2289	0.02	0.7748	−0.00	0.7958	0.14	0.2764
	rs9921541	chr16∶9,991,512–9,992,012	GT		−0.04	0.6482	0.03	0.6181	0.01	0.8932	−0.01	0.4727	0.07	0.5684
	rs4782039	chr16∶10,006,717–10,007,217	TC		−0.01	0.8343	0.01	0.9205	0.04	0.6066	0.00	0.8070	0.10	0.3853
	rs7190619	chr16∶10,078,874–10,079,374	GA		−0.01	0.9154	0.01	0.9256	−0.10	0.2778	0.02	0.3586	−0.08	0.5903
	rs9788936	chr16∶10,105,210–10,105,710	TC		0.05	0.5014	−0.03	0.6344	0.04	0.6484	0.01	0.7426	0.01	0.9558
	rs8057394	chr16∶10,115,238–10,115,738	GC		0.14	0.0525	0.04	0.4506	0.00	0.9916	0.01	0.3378	0.07	0.5246
	rs11644461	chr16∶10,120,640–10,121,140	TC		−0.13	0.0385	−0.02	0.6424	0.08	0.2276	−0.02	0.2136	−0.11	0.2730
	rs7192557	chr16∶10,123,219–10,123,719	GA		0.22	0.0159	0.08	0.2742	0.11	0.2443	0.00	0.9604	0.20	0.1877
	rs7206256	chr16∶10,196,673–10,197,173	AG		0.02	0.7845	0.02	0.7595	−0.00	0.9896	0.02	0.0919	0.00	0.9922
	rs1345423	chr16∶10,247,814–10,248,314	TG		−0.13	0.0421	0.01	0.8026	0.06	0.3536	−0.02	0.1442	−0.04	0.6942
	rs1650420	chr16∶10,268,080–10,268,580	GA		0.16	0.0193	−0.04	0.4499	−0.07	0.2912	0.02	0.1066	0.05	0.6514
**HSPG2**		chr1∶22,148,737–22,263,750												
	rs2270697	chr1∶22,167,738–22,168,238	GT		0.01	0.9194	−0.05	0.4670	−0.01	0.8797	0.01	0.5993	−0.14	0.2999
	rs2445142	chr1∶22,225,493–22,225,993	GC	[28]–[30]	−0.05	0.4292	0.08	0.1685	0.06	0.4036	0.01	0.6816	0.19	0.0998
	rs6698486	chr1∶22,243,773–22,244,273	CT		−0.10	0.2250	0.12	0.0760	−0.02	0.8130	−0.00	0.8494	0.25	0.0625
**DRD3**		chr3∶113,847,557–113,897,899		[25], [31]–[38]										
	rs9817063	chr3∶113,846,858–113,847,358	TC		−0.02	0.8083	−0.04	0.4632	−0.04	0.5922	0.02	0.2359	−0.01	0.9440
	rs2134655	chr3∶113,857,951–113,858,451	GA		0.02	0.7265	−0.05	0.4003	0.11	0.1409	0.03	0.0664	0.02	0.8868
	rs963468	chr3∶113,862,637–113,863,137	GA		−0.04	0.5906	0.03	0.5874	0.08	0.2021	−0.01	0.5302	−0.03	0.8098
	rs324035	chr3∶113,868,604–113,869,104	CA		0.00	0.9949	0.01	0.9005	−0.20	0.0392	−0.00	0.9592	0.12	0.4326
	rs3773678	chr3∶113,869,828–113,870,328	CT		−0.06	0.5968	0.05	0.5761	−0.21	0.0805	0.03	0.2130	0.18	0.3661
	rs167771	chr3∶113,876,025–113,876,525	AG		−0.06	0.5606	0.06	0.4746	−0.19	0.0512	0.00	0.9197	0.19	0.2265
	rs11721264	chr3∶113,879,154–113,879,654	GA		0.05	0.4688	−0.01	0.8877	−0.08	0.1930	−0.01	0.5338	−0.08	0.4382
	rs167770	chr3∶113,879,312–113,879,812	AG	[39]	0.05	0.3807	0.00	0.9367	−0.07	0.2411	−0.01	0.4278	−0.07	0.4798
	rs7633291	chr3∶113,886,818–113,887,318	TG	[39]	0.05	0.4719	0.00	0.9958	−0.05	0.5264	−0.02	0.1561	−0.08	0.4902
	rs1800828	chr3∶113,891,299–113,891,799	GC		0.01	0.8492	0.02	0.7948	−0.09	0.2275	−0.02	0.1701	0.02	0.8882
**DRD4**		chr11∶637,305–640,705		[40], [41]										
	rs3758653	chr11∶636,149–636,649	TC	[42]	−0.02	0.7043	−0.02	0.6344	−0.01	0.8712	0.00	0.6300	−0.08	0.2617
**HTR2C**		chrX:113,818,551–114,144,624		[25], [33], [35], [43]–[46]										
	rs569959	chrX:113,820,110–113,820,610	AG		0.01	0.8501	0.06	0.1606	−0.01	0.8138	−0.00	0.7311	0.11	0.1994
	rs17326429	chrX:113,826,117–113,826,617	GA		−0.07	0.3678	0.06	0.3194	−0.03	0.6436	−0.00	0.7743	0.14	0.2247
	rs12858300	chrX:113,897,163–113,897,663	GC		−0.03	0.6927	−0.10	0.1548	0.03	0.7543	−0.01	0.6958	−0.15	0.2892
	rs4911871	chrX:113,996,890–113,997,390	AG		−0.12	0.0634	0.02	0.7622	−0.06	0.3938	−0.01	0.6267	0.12	0.2648
	rs5946189	chrX:114,071,970–114,072,470	TC		0.03	0.5470	0.04	0.4574	−0.01	0.9251	0.02	0.0565	0.06	0.5314
	rs1801412	chrX:114,142,454–114,142,954	TG		−0.14	0.3456	−0.15	0.2627	−0.00	0.9991	−0.00	0.9149	−0.34	0.1571
**NQO1**		chr16∶69,743,304–69,760,533												
	rs1800566	chr16∶69,744,895–69,745,395	CT	[47]	−0.14	0.1126	0.01	0.8486	0.02	0.7939	0.02	0.3708	−0.11	0.4720

Sources: UCSC (GRCh37/hg19), NCBI, SNPedia, Genecards, CHIP Bioinformatics Tools.

a TD = tardive dyskinesia, PK = parkinsonism, AK = akathisia, TDt = tardive dystonia and PF = principal-factor.

In addition, variables possibly affecting risk were extracted from patients’ case notes (Text S1).

### Gene and Tag SNP Selection, DNA Extraction, Genotyping

Two 10 ml EDTA tubes of peripheral blood were drawn from participants, and genomic DNA was extracted from leucocytes by Autopure LS method (Qiangen) according to the manufacturer’s protocols.

The tag SNPs were selected using a web-based tool freely available on the internet (*SNPinfo Web Server*; http://www.niehs.nih.gov/snpinfo) [48] (Text S1).

### Statistical Analyses

#### Hardy weinberg equilibrium

Only SNPs were included in the analyses that were not significantly outside Hardy-Weinberg Equilibrium (HWE) (p>0.05) in (i) the complete control sample (for a dichotomous trait) or (ii) the complete study sample (for a continuous trait). For the six SNPs in the X-chromosomal *HTR2C* gene, departure from HWE was not calculated.

Departure from the HWE was calculated with the GENASS and GENHW procedures in the STATA statistical program [27] for (i) the dichotomously defined persistent forms of movement disorders separately in both patients (with one movement disorder) and controls (without that movement disorder). Case definition of a persistent movement disorder was based on 2 consecutive assessments over a period of minimally 3 months, and required that individuals met case definition criteria at two consecutive assessments (hereafter: persistent movement disorder), meeting the requirements of Schooler and Kane’s criteria for persistent movement disorder [49], and (ii) the combined group of patients and controls, as continuous measures cannot be separated in both patients and controls.

#### Association tests for single tag SNPs

Only continuous movement disorder outcomes were used, given that continuous measures better handle the variability of movement disorders and generate more statistical power than cut off points [50], [51]. Genotype and allele frequency comparisons were performed with multiple regression methods for continuous movement disorders, using the Armitage trend test, with the major allele (from our dataset of 168 selected white patients) as reference. The Armitage trend test assumes an additive effect by both alleles on the trait of interest, i.e. the mean effect on the trait by the heterozygous genotype (Major-Minor) is halfway the effects of the two homozygotes. (Major-Major and Minor-Minor).

#### Regression analyses

The regression analyses were conducted with movement disorder measures at a single assessment (hereafter: fluctuating movement disorder). The reason for this was that movement disorders constantly fluctuate over time, so that inclusion in the regression of their repeated single-occasion measures allowed for calculation of associations between one movement disorder with the other over time. As the study design comprised repeated measures nested in the same patient, clustering of observations in individuals needed to be corrected for. Therefore, multilevel random regression was used with the measurement occasion (baseline and two follow-ups) at level 1, and subjects at level 2, with the XTREG MLE routine of the STATA statistical program [27]. Associations with explanatory variables were expressed as beta coefficients representing the change of continuous movement disorder outcome with 1 unit change of the exposure variable.

Using the dataset of 168 selected white patients, associations with predictors were adjusted for *a priori*, movement-disorder specific covariates as follows (Bakker and colleagues, submitted) age was adjusted for in the model of TD and its subtypes; age and total antipsychotic use was adjusted for in the model of parkinsonism and its subtypes, and no covariates were introduced in the models of akathisia, tardive dystonia and the principal-factor.

Power calculations were performed using the Quanto program version 1.2.4 (http://hydra.usc.edu/gxe).

#### Correction for multiple testing

In order to correct for multiple testing of single SNP tests, the Simes modification of the Bonferroni multiple-testing procedure was performed to control the False Discovery Rate (FDR) [52]. Bonferroni correction is too conservative if tests are not independent of each other; in this case FDR represents a less conservative alternative. We used the MULTPROC procedure in the STATA statistical program [27] for FDR calculation, and then the SMILEPLOT procedure calling MULTPROC to build a smile plot. A smile plot summarizes a set of multiple analyses, similarly as a Cochrane forest plot summarizes a meta-analysis, and separates by reference line rejected and non-rejected p-values (on a reverse log scale against the corresponding parameter estimates).

#### Defined daily dose

Antipsychotic doses were converted to defined daily dose (DDD), for which we refer to our previous publication [19]. Anticholinergic medication was modeled as a dichotomous variable (yes/no).

## Results

### Sample Characteristics

Over the period of observation (mean = 1.1 years, SD = 0.64), of the 209 patients included at baseline, 207 participated in the study. One patient developed a brain tumor, another patient died after inclusion. All patients had a history of cumulative antipsychotic intake of minimally 1 year. Attrition rate was low at 9.8% over a 4-year period.

Of the 207 patients, with chronic psychiatric illness requiring long-term admission, 199 participated in the genetic study. To prevent ethnic stratification resulting in spurious associations owing to differences in allele frequencies and risk of movement disorders, only white patients, representing the most prevalent group (168 = 84.4%), were included in the analysis. At baseline, mean age expressed in years was 48.8 (SD 12.4); men 48.6 (SD 12.5) and women 49.1 (SD 12.2). Age at first admission, expressed in years, was 25.1 (SD 8.8); men 23.7 (SD 7.8) and women 27.1 (SD 9.7). The total duration of admission, expressed in years, was 23.4 (SD 12.9), men 24.4 (SD 12.5) and women 22.0 (SD 13.4). Diagnoses according to DSM-IV Axis I as defined above were: schizophrenia 112 (66.7%), psychosis 9 (5.4%), affective disorder 27 (16.1%), other Axis I diagnosis 11 (6.6%) and no Axis I diagnosis 9 (5.4%).

### Association Analyses with Tag SNPs

The following SNPs were excluded from analysis, due to deviation from HWE: all movement disorders – rs6698486, rs11721264, rs167770, rs3758653 and rs1800566, as well as controls, TD – rs10845838, rs7206256 and rs7633291; orofacial dyskinesia – rs10845838 and rs2445142; limb truncal dyskinesia – rs7633291; parkinsonism, rest tremor and bradykinesia – rs2445142. In addition, rs12300851 was removed as it contained only A alleles.

**Figure 1:**
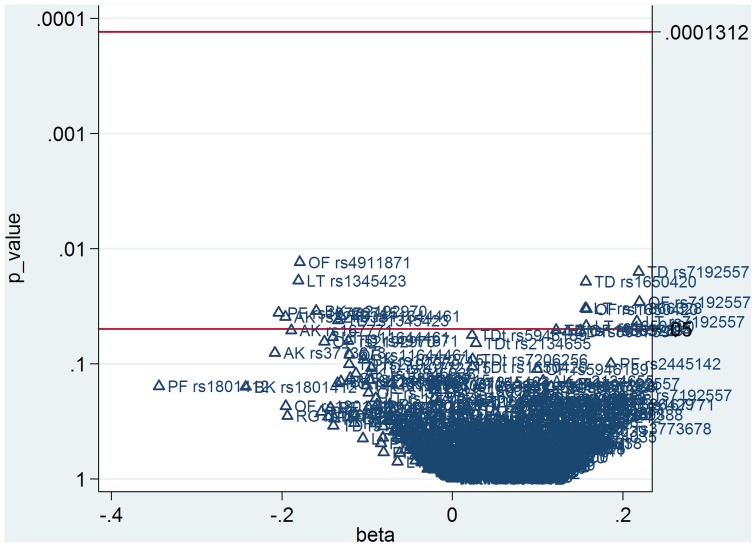
Smile plot summarizing set of multiple analyses after Simes correction for multiple testing of tag SNPs without HWE deviation. Corresponding p-values (on a reverse log scale against the corresponding parameter estimates). TD = tardive dyskinesia, OF = orofacial dyskinesia, LT = limb truncal dyskinesia, PK = parkinsonism, RT = rest tremor, RG = rigidity, BK = bradykinesia, AK = akathisia,TDt = tardive dystonia.

The (multilevel) regression yielded significant coefficients, after adjustment for age, between TD and rs1345423 (B = −0.13, p = 0.0421), rs7192557 (B = 0.22, p = 0.0159), rs1650420 (B = 0.16, p = 0.0193), as well as rs11644461 (B = −0.13, p = 0.0385); between orofacial dyskinesia and rs7192557 (B = 0.22, p = 0.0291), rs1650420 (B = 0.16, p = 0.0336), as well as rs4911871 (B = −0.18, p = 0.0131); between limb truncal dyskinesia and rs1345423 (B = −0.18, p = 0.0190), rs7192557 (B = 0.22, p = 0.0430), rs1650420 (B = 0.16, p = 0.0471), as well as rs11866328 (B = 0.16, p = 0.0330). After adjustment for age and total DDD equivalents, associations were apparent between bradykinesia and rs2192970 (B = −0.16, p = 0.0349). Without adjustment, associations were apparent between akathisia and rs324035 (B = −0.20, p = 0.0392), as well as the principal-factor and rs10772715 (B = −0.20, p = 0.0362). After Simes correction for multiple testing of the above mentioned analyses, the number of rejected p-values was zero, with a corrected overall critical p-value of 0.00013 (Figure 1).

Power calculations showed that our sample was insufficiently powered (0.05) to identify the betas from our regressions, which were between −0.34 and 0.25.

## Discussion

In a population with chronic mental illness, various tag SNPs in 7 candidate genes (*GRIN2B*, *GRIN2A*, *HSPG2*, *DRD3*, *DRD4*, *HTR2C*, and *NQO1*) reached nominally significant (p≤0.05) associations with drug-induced movement disorders. However, after controlling for multiple testing, our findings suggest that these tag SNPs are not associated with a susceptibility to movement disorders.

Another reason for the inconclusive findings could be explained by the fact that in a naturalistic setting it is possible to evaluate the overall impact of pharmacogenetic signals in the presence of a host of real-life variables that can override pharmacogenetic variation. The fact we did not observe a significant association may also attest to the possibility that each gene makes a small contribution that is often diluted or overridden by environmental and clinical variations.

### Limitations

**Figure 2:**
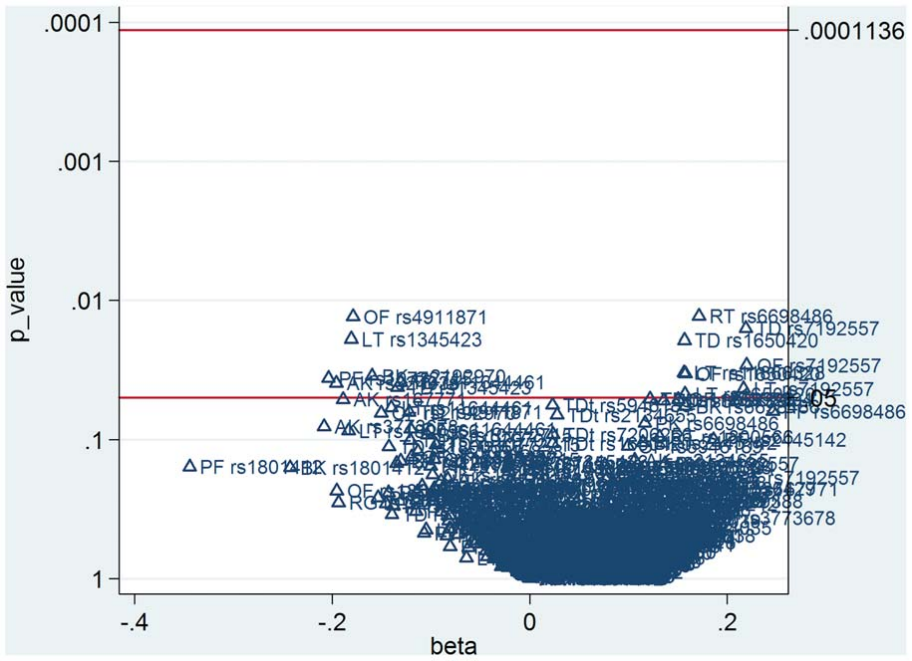
Smile plot summarizing set of multiple analyses after Simes correction for multiple testing of all tag SNPs. Corresponding p-values (on a reverse log scale against the corresponding parameter estimates). TD = tardive dyskinesia, OF = orofacial dyskinesia, LT = limb truncal dyskinesia, PK = parkinsonism, RT = rest tremor, RG = rigidity, BK = bradykinesia, AK = akathisia,TDt = tardive dystonia.

This study had limitations, for which we refer to our previous publications [19], [53] (Bakker and colleagues, submitted) as well point to additional limitations. First, some authors may argue that the SNPs with HWE deviation should not be excluded from the analyses, as SNPs in HWE could in reality be also out of HWE owing to lack of power. Therefore, we performed a *post-hoc* analysis with all SNPs, i.e., also those deviating from HWE, which resulted in one extra nominal significant result, which also did not survive Simes correction for multiple testing (Figure 2). Second, the choice to genotype tag SNPs in the candidate genes, and not only the specific SNPs that were previously associated with movement disorders, increased the multiple testing burden. However, tag SNPs are a relatively small set of common polymorphisms [54] in the candidate genes providing maximum information; therefore, they may reveal new (unknown) SNPs in linkage disequilibrium related to movement disorders. Third, the inclusion of patients with various psychiatric illnesses, not only schizophrenia, may result in spurious associations.

### Strengths

We refer to our previous publications [19], [53] (Bakker and colleagues, submitted). The importance of repeated measures should be noted, as case definition of repeated measures, rather than a single cross-sectional measure, for continuous movement disorders better reflects the continuously fluctuating nature in time of both acute and chronic movement disorders, and therefore may represent a more suitable standard in future research. To the best of our knowledge only few papers in the literature address this issue.

As the sample size of the current study is small and previous studies show inconsistent results, definite conclusions cannot be made. Yet the question is how to interpret these results. In our opinion, the findings of weak genetic signals need to be replicated in larger study samples, preferably in longitudinal studies which take the fluctuating course of movement disorders and gene-environment interactions into account [55], [56].

Even though the current study is inconclusive, the importance of negative findings should not be underestimated as they can inform future meta-analysis, which otherwise might be biased by excess significance bias occasioned by selection of positive studies for publication. The pioneering meta-analyses of Lerer and colleagues [31], [57] showed that with meta-analysis, synthesis of association studies for TD can be accomplished; it was shown that small effects may point to the involvement of other genes in the development of TD. In recent years, genome-wide association studies (GWASs) revealed several SNPs related to movement disorders including tardive dyskinesia [29], [30], [58], [59] and parkinsonism [60], [61]. We further refer to our previous publication [53].

All in all, it seems legitimate to conclude that future research projects into antipsychotic-induced movement disorders may take advantage of a new perspective on common pathways of movement disorders. *Directed acyclic graphs* (DAGs, also known as “causal diagrams” or “causal pathways”) allow pathways to be visualized by analytical graphs and conceptualize the relationships between all of the important variables (e.g., schizophrenia, movement disorder, antipsychotics, genes, etc.) as a precise theoretical model. DAGs depict explicitly, in an easy and flexible way, confounding effects (“backdoor paths”) and collider effects (two causal pathways). The latter is important as adjusting for colliders creates confounding [62] ^(p161)^ [63] ^(p183)^. Rothman’s sufficient-component cause model (or sufficient-cause model) permits the postulation of different *sufficient causes* comprising a collection of collaborating risk factors (also known as “causal components”) “sufficient to produce the disease in the individual” [63] ^(p8)^. This model can also identify *proximal* (biological markers of risk), *intermediate,* and *distal* sufficient causes, hence describing a chain of causality [62] ^(p379)^.

The sufficient-component cause model shows that risk factors in complex diseases plausibly interact non-additively, since these risk factors may be neither necessary nor sufficient to produce disease, and frequently co-participate in similar pathways [63] ^(p81)^ [64], [65]. Although in psychiatry there is great interest in these interactions (gene-environment, environment-environment and gene-gene), concerns are being raised about the correct underlying interaction model (additive versus multiplicative), as are doubts about the recent interpretation of pathogenesis increasingly identified in a growing number of interaction studies [66].

New research designs to help understand gene-environment causality are in progress. For example, contrary to between-subject cross-sectional designs, longitudinal within-subject (longitudinal and multilevel) designs with repeated assessments [67] are being implemented. They have the advantage of being free of between-subject confounding [68], may reveal the dynamics of behavior (movements), and therefore elucidate gene-environment causality. Of importance are the so called momentary assessment tools [69], examples being the Experience Sampling Method (ESM) by Csikszentmihalyi and Larson [70] and ‘PsyMate’ by Myin-Germeys [71].

In addition, future research may be served by data-intensive science that concentrates on sharing and integrating selected data sets [72]. As rightly stated by Chalise and colleagues [73], new sets of integrative genomic approach, i.e., the collection of different types of genomic data, do more justice to the complexity of drug-related phenotypes, than most of the existing “naïve one-at-a-time analysis approach” which overlooks this complexity. Important initiatives are (i) the EUropean network of national schizophrenia networks studying Gene-Environment Interactions (EU-GEI, www.eu-gei.eu) and (ii) the Network of Investigator Networks sponsored by the global Human Genome Epidemiology Network (HuGENet, www.cdc.gov/genomics/hugenet) [74]. An important issue concerns the identification of significant SNPs in the genome, e.g. from genome-wide association studies (GWASs) [75].

In conclusion, the findings suggest that selected tag SNPs are not associated with a susceptibility to movement disorders. However, replication is needed in larger study samples, preferably in longitudinal studies which take the fluctuating course of movement disorders and gene-environment interactions into account. The use of intermediate phenotypes, for example, laboratory based phenotypes [76], or more accurate measures of movement disorders, for example instrument measurement of lingual force variability as proposed by Koning and colleagues [77], which may represent a powerful alternative since instrument measurement detects subclinical movement disorders and is highly reliable. Moreover, (pharmaco) genetic studies may help elucidate common pathways in the development of movement disorders. Future research on movement disorders may be served by the inclusion of all four movement disorders, as performed in the current study, since they may represent pleiotropic effects from (partly) shared genetic factors [78].

## Supporting Information

Table S1 **Selected 45 tag SNPs for multilevel regression of continuous movement disorders (subtypes).**
(DOC)

Text S1 **Supporting information about the 7 candidate genes.**
(DOC)
