# Supplementary material for: Antipsychotic-Induced Movement Disorders in Long-Stay Psychiatric Patients and 45 Tag SNPs in 7 Candidate Genes: A Prospective Study
Source: PLoS One. 2012 Dec 4;7(12):e50970. doi: 10.1371/journal.pone.0050970 (PMC3514178; doi:10.1371/journal.pone.0050970)
Supplement: Table S1 — Selected 45 tag SNPs for multilevel regression of continuous movement disorders (subtypes). (DOC) [file pone.0050970.s001.doc]

**Table S1** *Selected 45 tag SNPs for multilevel regression of continuous movement disorders (subtypes)*

| **Gene** | **Tag SNP** | **Chromosome Position** | **Alleles Public** | **OF^a^** |  | **LT^a^** |  | **RT^a^** |  | **RG^a^** |  | **BK^a^** |  |
| --- | --- | --- | --- | --- | --- | --- | --- | --- | --- | --- | --- | --- | --- |
|  |  |  |  |  |  |  |  |  |  |  |  |  |  |
|  |  |  | Major/Minor | *Beta* | *p-value* | *Beta* | *p-value* | *Beta* | *p-value* | *Beta* | *p-value* | *Beta* | *p-value* |
| **GRIN2B** |  | chr12:13,714,410-14,133,022 |  |  |  |  |  |  |  |  |  |  |  |
|  | rs1805481 | chr12:13,763,205-13,763,705 | AC | -0.04 | 0.5915 | -0.03 | 0.7296 | 0.01 | 0.9092 | 0.02 | 0.7402 | 0.09 | 0.2115 |
|  | rs7313149 | chr12:13,828,037-13,828,537 | TC | 0.07 | 0.3538 | -0.05 | 0.5631 | -0.00 | 0.9328 | 0.05 | 0.5236 | 0.06 | 0.4324 |
|  | rs2192970 | chr12:13,836,063-13,836,563 | CT | -0.15 | 0.0643 | -0.08 | 0.3507 | 0.08 | 0.2240 | -0.07 | 0.3895 | -0.16 | 0.0349 |
|  | rs2300242 | chr12:13,840,047-13,840,547 | TA | 0.03 | 0.7308 | -0.02 | 0.8265 | 0.03 | 0.6145 | -0.03 | 0.6929 | 0.08 | 0.2515 |
|  | rs10845838 | chr12:13,894,146-13,894,646 | GA | 0.03 | 0.7096 | 0.09 | 0.2742 | -0.03 | 0.5996 | -0.04 | 0.6204 | 0.02 | 0.7801 |
|  | rs12300851 | chr12:13,968,155-13,968,655 | TC | - | - | - | - | - | - | - | - | - | - |
|  | rs220599 | chr12:13,975,048-13,975,548 | GA | 0.01 | 0.8738 | -0.06 | 0.3940 | 0.05 | 0.3622 | -0.03 | 0.6105 | 0.06 | 0.3732 |
|  | rs10772715 | chr12:14,037,753-14,038,253 | GA | -0.10 | 0.1388 | -0.09 | 0.1993 | -0.03 | 0.5804 | -0.04 | 0.5428 | -0.10 | 0.0924 |
|  | rs12827536 | chr12:14,095,907-14,096,407 | CT | -0.05 | 0.4325 | -0.03 | 0.7106 | 0.04 | 0.4156 | -0.00 | 0.9618 | 0.02 | 0.8070 |
|  |  |  |  |  |  |  |  |  |  |  |  |  |  |
| **GRIN2A** |  | chr16:9,847,265-10,276,263 |  |  |  |  |  |  |  |  |  |  |  |
|  | rs11866328 | chr16:9,862,306-9,862,806 | GT | 0.10 | 0.1584 | 0.16 | 0.0330 | -0.02 | 0.7453 | -0.04 | 0.5871 | -0.02 | 0.7812 |
|  | rs11646587 | chr16:9,873,069-9,873,569 | GA | -0.01 | 0.8955 | -0.06 | 0.4427 | -0.03 | 0.6147 | -0.05 | 0.5323 | -0.07 | 0.3152 |
|  | rs7196095 | chr16:9,885,582-9,886,082 | TC | 0.04 | 0.5529 | 0.00 | 0.9536 | -0.04 | 0.4543 | -0.04 | 0.5447 | -0.01 | 0.9279 |
|  | rs8049651 | chr16:9,943,416-9,943,916 | CT | 0.08 | 0.3421 | 0.03 | 0.7615 | -0.05 | 0.4205 | -0.04 | 0.6465 | 0.06 | 0.4255 |
|  | rs9989388 | chr16:9,965,889-9,966,389 | CT | -0.03 | 0.7519 | -0.04 | 0.7011 | 0.04 | 0.5962 | 0.03 | 0.6959 | 0.10 | 0.2029 |
|  | rs9921541 | chr16:9,991,512-9,992,012 | GT | -0.03 | 0.7641 | -0.04 | 0.6282 | 0.02 | 0.7763 | -0.03 | 0.6773 | 0.05 | 0.5426 |
|  | rs4782039 | chr16:10,006,717-10,007,217 | TC | -0.07 | 0.3817 | 0.05 | 0.5471 | -0.01 | 0.8785 | -0.01 | 0.9464 | 0.02 | 0.7755 |
|  | rs7190619 | chr16:10,078,874-10,079,374 | GA | 0.00 | 0.9790 | -0.02 | 0.8197 | 0.05 | 0.5052 | 0.03 | 0.7665 | -0.01 | 0.8938 |
|  | rs9788936 | chr16:10,105,210-10,105,710 | TC | 0.02 | 0.8278 | 0.09 | 0.3192 | -0.06 | 0.3738 | -0.01 | 0.9209 | -0.02 | 0.7592 |
|  | rs8057394 | chr16:10,115,238-10,115,738 | GC | 0.15 | 0.0514 | 0.12 | 0.1586 | -0.04 | 0.4757 | 0.09 | 0.2150 | 0.08 | 0.2732 |
|  | rs11644461 | chr16:10,120,640-10,121,140 | TC | -0.12 | 0.0840 | -0.14 | 0.0590 | 0.04 | 0.4957 | -0.04 | 0.5937 | -0.05 | 0.4055 |
|  | rs7192557 | chr16:10,123,219-10,123,719 | GA | 0.22 | 0.0291 | 0.22 | 0.0430 | -0.04 | 0.6219 | 0.11 | 0.2469 | 0.14 | 0.1487 |
|  | rs7206256 | chr16:10,196,673-10,197,173 | AG | 0.00 | 0.9603 | 0.04 | 0.6505 | -0.01 | 0.8298 | 0.09 | 0.2029 | 0.01 | 0.8977 |
|  | rs1345423 | chr16:10,247,814-10,248,314 | TG | -0.10 | 0.1793 | -0.18 | 0.0190 | 0.04 | 0.4397 | -0.01 | 0.8527 | 0.00 | 0.9468 |
|  | rs1650420 | chr16:10,268,080-10,268,580 | GA | 0.16 | 0.0336 | 0.16 | 0.0471 | -0.05 | 0.3649 | -0.08 | 0.3053 | -0.03 | 0.6849 |
|  |  |  |  |  |  |  |  |  |  |  |  |  |  |
| **HSPG2** |  | chr1:22,148,737-22,263,750 |  |  |  |  |  |  |  |  |  |  |  |
|  | rs2270697 | chr1:22,167,738-22,168,238 | GT | -0.03 | 0.7653 | 0.06 | 0.5323 | 0.04 | 0.5252 | -0.04 | 0.6819 | -0.10 | 0.2393 |
|  | rs2445142 | chr1:22,225,493-22,225,993 | GC | -0.07 | 0.3673 | -0.04 | 0.6484 | 0.08 | 0.1891 | -0.05 | 0.5362 | 0.11 | 0.1050 |
|  | rs6698486 | chr1:22,243,773-22,244,273 | CT | -0.11 | 0.2150 | -0.08 | 0.3847 | 0.17 | 0.0130 | -0.10 | 0.2559 | 0.16 | 0.0576 |
|  |  |  |  |  |  |  |  |  |  |  |  |  |  |
| **DRD3** |  | chr3:113,847,557-113,897,899 |  |  |  |  |  |  |  |  |  |  |  |
|  | rs9817063 | chr3:113,846,858-113,847,358 | TC | -0.03 | 0.6843 | 0.01 | 0.9409 | -0.02 | 0.6709 | -0.02 | 0.8236 | -0.05 | 0.4257 |
|  | rs2134655 | chr3:113,857,951-113,858,451 | GA | -0.02 | 0.8331 | 0.07 | 0.3726 | -0.05 | 0.4022 | -0.06 | 0.4244 | -0.04 | 0.5832 |
|  | rs963468 | chr3:113,862,637-113,863,137 | GA | 0.01 | 0.9297 | -0.09 | 0.2398 | 0.05 | 0.3587 | -0.01 | 0.8316 | 0.03 | 0.6381 |
|  | rs324035 | chr3:113,868,604-113,869,104 | CA | -0.03 | 0.7986 | 0.03 | 0.7646 | -0.03 | 0.7237 | -0.02 | 0.8174 | 0.03 | 0.7673 |
|  | rs3773678 | chr3:113,869,828-113,870,328 | CT | -0.03 | 0.8209 | -0.11 | 0.4466 | 0.03 | 0.7807 | -0.01 | 0.9626 | 0.07 | 0.5482 |
|  | rs167771 | chr3:113,876,025-113,876,525 | AG | -0.08 | 0.4382 | -0.03 | 0.8165 | 0.00 | 0.9582 | 0.05 | 0.6465 | 0.08 | 0.4284 |
|  | rs11721264 | chr3:113,879,154-113,879,654 | GA | 0.04 | 0.5668 | 0.06 | 0.4369 | -0.02 | 0.6977 | 0.07 | 0.3337 | -0.03 | 0.6925 |
|  | rs167770 | chr3:113,879,312-113,879,812 | AG | 0.05 | 0.4647 | 0.06 | 0.3770 | -0.01 | 0.9114 | 0.07 | 0.2728 | -0.01 | 0.8245 |
|  | rs7633291 | chr3:113,886,818-113,887,318 | TG | 0.08 | 0.3369 | 0.03 | 0.7380 | -0.01 | 0.8322 | 0.06 | 0.4838 | -0.01 | 0.8919 |
|  | rs1800828 | chr3:113,891,299-113,891,799 | GC | 0.05 | 0.4931 | -0.03 | 0.7112 | -0.04 | 0.5017 | 0.06 | 0.4535 | 0.03 | 0.6648 |
|  |  |  |  |  |  |  |  |  |  |  |  |  |  |
| **DRD4** |  | chr11:637,305-640,705 |  |  |  |  |  |  |  |  |  |  |  |
|  | rs3758653 | chr11:636,149-636,649 | TC | -0.01 | 0.8876 | -0.03 | 0.5476 | 0.03 | 0.4917 | 0.00 | 0.9830 | -0.04 | 0.3449 |
|  |  |  |  |  |  |  |  |  |  |  |  |  |  |
| **HTR2C** |  | chrX:113,818,551-114,144,624 |  |  |  |  |  |  |  |  |  |  |  |
|  | rs569959 | chrX:113,820,110-113,820,610 | AG | 0.04 | 0.4266 | -0.04 | 0.5320 | 0.05 | 0.2614 | 0.02 | 0.7102 | 0.08 | 0.1525 |
|  | rs17326429 | chrX:113,826,117-113,826,617 | GA | -0.12 | 0.1335 | 0.00 | 0.9572 | 0.06 | 0.3210 | -0.02 | 0.8136 | 0.08 | 0.2801 |
|  | rs12858300 | chrX:113,897,163-113,897,663 | GC | -0.09 | 0.3296 | 0.05 | 0.6440 | -0.01 | 0.9112 | -0.13 | 0.1482 | -0.13 | 0.1424 |
|  | rs4911871 | chrX:113,996,890-113,997,390 | AG | -0.18 | 0.0131 | -0.05 | 0.5285 | 0.02 | 0.7033 | -0.02 | 0.7997 | 0.02 | 0.7669 |
|  | rs5946189 | chrX:114,071,970-114,072,470 | TC | 0.10 | 0.1115 | -0.05 | 0.4537 | 0.02 | 0.6628 | 0.02 | 0.7279 | 0.04 | 0.4561 |
|  | rs1801412 | chrX:114,142,454-114,142,954 | TG | -0.20 | 0.2339 | -0.06 | 0.7126 | 0.02 | 0.8899 | -0.19 | 0.2843 | -0.24 | 0.1591 |
|  |  |  |  |  |  |  |  |  |  |  |  |  |  |
| **NQO1** |  | chr16:69,743,304-69,760,533 |  |  |  |  |  |  |  |  |  |  |  |
|  | rs1800566 | chr16:69,744,895-69,745,395 | CT | -0.11 | 0.2369 | -0.18 | 0.0863 | 0.13 | 0.0955 | 0.05 | 0.5845 | -0.06 | 0.5328 |

Sources: UCSC (GRCh37/hg19), NCBI, SNPedia, Genecards, CHIP Bioinformatics Tools

^a^ OF=orofacial dyskinesia, LT=limb truncal dyskinesia, RT=rest tremor, RG=rigidity, BK=bradykinesia
