# Supplementary material for: Antipsychotic-Induced Movement Disorders in Long-Stay Psychiatric Patients and 45 Tag SNPs in 7 Candidate Genes: A Prospective Study
Source: PLoS One. 2012 Dec 4;7(12):e50970. doi: 10.1371/journal.pone.0050970 (PMC3514178; doi:10.1371/journal.pone.0050970)
Supplement: Text S1 — Supporting information about the 7 candidate genes. (DOC) [file pone.0050970.s002.doc]

Antipsychotic-induced Movement Disorders in Long-Stay Psychiatric Patients and 45 tag SNPs in 7 candidate genes:

A Prospective Study

P. Roberto Bakker^a^, M.D. Ph.D.

Asmar F.Y. Al Hadithy^b^, Ph.D. Pharm.D.

Najaf Amin^c^, Ph.D.

Cornelia M. van Duijn^c^, Ph.D.

Jim van Os^d,e^, M.D. Ph.D.

Peter N. van Harten^a,d^, M.D. Ph.D.

Supporting Text S1

^a^Psychiatric Centre GGZ Centraal, Amersfoort, The Netherlands

^b^Hospital Pharmacy, Erasmus MC, Rotterdam, The Netherlands

^c^Department of Epidemiology, Erasmus MC, Rotterdam, The Netherlands

^d^Department of Psychiatry and Psychology, South Limburg Mental Health Research and Teaching Network, EURON, Maastricht University Medical Centre, Maastricht, The Netherlands

^e^King's College London, King's Health Partners, Department of Psychosis Studies, Institute of Psychiatry, London, United Kingdom

Corresponding author:

P.R. Bakker

c/o Mrs. H. Brouwer

Psychiatric Centre GGZ Centraal, Amersfoort, The Netherlands, Medisch Centrum

PO BOX 3051

3800 DB Amersfoort

The Netherlands

Tel.: +31 33 4609568

Fax: +31 33 4609557

e-mail: robertobakker@gmail.com

## Genes

A systematic literature review was conducted of the literature published between 1976 and July 2012, with the help of Medline, EMBASE and PsychINFO using key words (genetic) polymorphism(s), tardive dyskinesia, extrapyramidal (syndrome/disorder), *drug-induced,* *antipsychotic(s), adverse effect*/event, schizophrenia. In addition, all relevant references cited in these articles were also retrieved.

***S1.1 Tardive dyskinesia***

The dopaminergic and serotonergic systems of neurotransmission have been implicated in the development of movement disorders.

Genes involved in dopaminergic signaling, possibly associated with the development of tardive dyskinesia (TD), include those coding for: (i) Dopamine 3 receptor (*DRD3*), for which we refer to our previous publication [1]. A recent meta-analysis in Asian populations did not find an association between Ser9Gly (rs6280) in *DRD3* and TD [2]; and (ii) Dopamine 4 receptor (*DRD4*), as clozapine’s atypical action may be attributed to its 10 times stronger dopamine 4 receptor affinity than dopamine 2 or 3 [3–7], with evidence for an association between 4 tag SNPs haplotype (rs3758653, rs916457, rs762502 and rs11246226) and TD in Caucasian men, but not the separate SNPs or the exon 3 variable number tandem-repeat (exon 3 VNTR) in the combined sample of men and women [8]. A former study in an North Indian population [9] showed a significant association between the 120bp duplication allele in *DRD4* and TD, but not the exon 3 VNTR or -521 C/T ([rs1800955](http://www.ncbi.nlm.nih.gov/SNP/snp_ref.cgi?rs=rs1800955)). A study in Korean patients [10] did not find evidence for an association between [rs1800955](http://www.ncbi.nlm.nih.gov/SNP/snp_ref.cgi?rs=rs1800955) polymorphism and TD. Another study showed association between the 'short' variant of the exon 3 VNTR in *DRD4* and TD at trend significance [11]. Segman and colleagues [12] did not show an association between the 120bp duplication allele nor the exon 3 VNTR and TD.

The serotonin 2C receptor (*HTR2C*) gene has been studied in TD, for which we refer to our previous publication [1].

*Oxidative stress-mediated neurotoxic damage*

The neuronal degeneration hypothesis, in the context of neuronal death or neurotoxicity, has been proposed as an alternative to the ‘dopamine supersensitivity hypothesis’ in the development of TD [13,14]. One argument for the neuronal degeneration hypothesis is that the supersensitivity hypothesis may not fit the clinical course of TD because: (i) although hypersensitivity seems to be a universal response to D2-receptor antagonists, not all patients develop TD, (ii) TD tends to display an irreversible course, whereas dopamine supersensitivity diminishes gradually upon cessation of antipsychotics, and (iii) the risk for TD is markedly elevated with age, but the dopamine supersensitivity response may be dampened with increasing age [15].

Genes involved in the protection of neurotoxicity, possibly associated with the development of TD, include those coding for: (i) NAD(P)H:quinone oxidoreductase 1 (NQO1; also known as NAD(P)H dehydrogenase, quinone 1) [16], implicated against neurotoxic stress, albeit with no evidence in a recent study with subsequent meta-analysis for an association between Pro187Ser (rs1800566, C609T) and TD [17], (ii) glutamate receptor, ionotropic, N-methyl D-aspartate 2B (*GRIN2B*), albeit without evidence for an association between three polymorphisms (T-200G, C366G and C2664T) in *GRIN2B* and TD [18]. Another study did not find a significant association between 9 polymorphisms (same as in the current study) in *GRIN2B* and TD [19], (iii) glutamate receptor, ionotropic, N-methyl D-aspartate 2A (*GRIN2A*), without evidence for an association between 15 polymorphisms (same as in the current study) in *GRIN2A* and TD [19].

More details on this topic can be found in the recent extensive review by Lee and Kang [20].

*HSPG2 (heparan sulfate proteoglycan 2) gene*

Two studies showed a significant association between rs2445142 in *HSPG2* and TD [21,22], a SNP originally found in a genome-wide study performed by the latter group [23].

***S1.2. Parkinsonism***

The pharmacological explanation of antipsychotic-induced parkinsonism (AIP) is antagonism of the nigrostriatal dopamine D2 receptor [24,25].

Dopamine 4 receptor (*DRD4*) as explanation for lower AIP in atypical action is mentioned by Seeman and colleagues [4,5].

***S1.3 Akathisia***

An association between the Ser9Gly polymorphism in *DRD3* and the risk to develop akathisia has been reported [26].

***S1.4 Tardive dystonia***

To the best of our knowledge, studies between genes coding for *GRIN2B*, *GRIN2A*, *HSPG2*, *DRD3*, *DRD4*, *HTR2C*, and *NQO1* and tardive dystonia have not been performed yet.

***S1.5 Gene-gene interactions***

We refer to our previous publication [1].

## Variables

In addition, variables possibly affecting risk were extracted from patients’ case notes including age, sex, BMI, self-reported handedness, diagnosis according to DSM-IV, ethnic group (classified as white and non-white), duration of hospitalization and history of electroconvulsive therapy (ECT). Negative symptoms were rated using the negative symptom subscale of the Positive and Negative Symptom Severity (PANSS) scale [27]. The MINI sections for alcohol and drug use were administered, and information on tobacco intake (yes/no, number of cigarettes, cigars, etc; descriptors such as ’light’, ‘mild’, ‘heavy’ and ‘normal’ use of tobacco) was collected. At baseline and at each follow-up assessment, current use of antipsychotic and anticholinergic medication was collected, and the global symptom rating of the Clinical Global Impression – Schizophrenia severity of illness (CGI-SCH SI) scale was completed. All clinical assessments were carried out by a psychiatrist (PRB). Information on current use of the above medication was collected from the hospital and outpatient pharmacy databases.

The diagnosis ‘schizophrenia’ hereafter refers to DSM-IV codes 295.30, 295.10, 295.20, 295.90, 295.60, 295.70, and other diagnoses of ‘psychotic disorder’ to 295.40, 297.1, 298.8, 298.9.

## Gene and tag SNP selection, DNA extraction, Genotyping

Two 10 ml EDTA tubes of peripheral blood were drawn from participants, and genomic DNA was extracted from leucocytes by Autopure LS method (Qiangen) according to the manufacturer’s protocols.

The tag SNPs were selected using a web-based tool freely available on the internet (*SNPinfo Web Server*; http://www.niehs.nih.gov/snpinfo) [28] (Text S1). The following criteria have been applied for the selection of the tag SNPs: localization in the gene including 1000 bp upstream and downstream (5’- and 3’ flanking regions), LD threshold=0.8, Minor Allele Frequency (MAF) ≥ 0.1, Maximal distance between SNPs for calculation of LD = 250000 bp, Genotype data = “European” [dbSNP].

In the case of *GRIN2B* and *GRIN2A* our search query resulted in too many tag SNPs. We have therefore limited our selection to only those tag SNPs that capture at least 10 other SNPs.

After the selection process, we have genotyped 48 tag SNPs in the 7 candidate gene regions by the use of Veracode (GoldenGate) Assay (Illumina, San Diego, California, USA). Two of the selected tag SNPs in *HTR2C* (rs6318 and rs3813929) and rs6280 in *DRD3* have already been analyzed (TaqMan® SNP Genotyping Assays method, Applied Biosystems, Foster City, California, USA) in a previous study [1] and were therefore excluded from the analyses.

# References

1. Bakker PR, Bakker E, Amin N, van Duijn CM, van Os J, et al. (2012) Candidate gene-based association study of antipsychotic-induced movement disorders in long-stay psychiatric patients: a prospective study. PLoS ONE 7: e36561. 10.1371/journal.pone.0036561 [doi];PONE-D-12-00737 [pii].

2. Utsunomiya K, Shinkai T, Sakata S, Yamada K, Chen HI, et al. (2012) Genetic association between the dopamine D3 receptor gene polymorphism (Ser9Gly) and tardive dyskinesia in patients with schizophrenia: a reevaluation in East Asian populations. Neurosci Lett 507: 52-56. S0304-3940(11)01572-2 [pii];10.1016/j.neulet.2011.11.050 [doi].

3. Wong AH, Van Tol HH (2003) The dopamine D4 receptors and mechanisms of antipsychotic atypicality. Prog Neuropsychopharmacol Biol Psychiatry 27: 1091-1099.

4. Seeman P, Corbett R, Van Tol HH (1997) Atypical neuroleptics have low affinity for dopamine D2 receptors or are selective for D4 receptors. Neuropsychopharmacology 16: 93-110. S0893133X9600187X [pii];10.1016/S0893-133X(96)00187-X [doi].

5. Seeman P, Corbett R, Van Tol HH (1998) Dopamine D4 receptors may alleviate antipsychotic-induced parkinsonism. Adv Pharmacol 42: 478-482.

6. Van Tol HH, Wu CM, Guan HC, Ohara K, Bunzow JR, et al. (1992) Multiple dopamine D4 receptor variants in the human population. Nature 358: 149-152.

7. Van Tol HH, Bunzow JR, Guan HC, Sunahara RK, Seeman P, et al. (1991) Cloning of the gene for a human dopamine D4 receptor with high affinity for the antipsychotic clozapine. Nature 350: 610-614. 10.1038/350610a0 [doi].

8. Zai CC, Tiwari AK, Basile V, De Luca V, Muller DJ, et al. (2009) Association study of tardive dyskinesia and five DRD4 polymorphisms in schizophrenia patients. Pharmacogenomics J 9: 168-174.

9. Srivastava V, Varma PG, Prasad S, Semwal P, Nimgaonkar VL, et al. (2006) Genetic susceptibility to tardive dyskinesia among schizophrenia subjects: IV. Role of dopaminergic pathway gene polymorphisms. Pharmacogenet Genomics 16: 111-117.

10. Lee HJ, Kang SG, Choi JE, Paik JW, Kim YK, et al. (2007) No association between dopamine D4 receptor gene -521 C/T polymorphism and tardive dyskinesia in schizophrenia. Neuropsychobiology 55: 47-51. 000103576 [pii];10.1159/000103576 [doi].

11. Lattuada E, Cavallaro R, Serretti A, Lorenzi C, Smeraldi E (2004) Tardive dyskinesia and DRD2, DRD3, DRD4, 5-HT2A variants in schizophrenia: an association study with repeated assessment. Int J Neuropsychopharmacol 7: 489-493.

12. Segman RH, Goltser T, Heresco-Levy U, Finkel B, Shalem R, et al. (2003) Association of dopaminergic and serotonergic genes with tardive dyskinesia in patients with chronic schizophrenia. Pharmacogenomics J 3: 277-283.

13. Andreassen OA, Jorgensen HA (2000) Neurotoxicity associated with neuroleptic-induced oral dyskinesias in rats. Implications for tardive dyskinesia? Prog Neurobiol 61: 525-541. S0301-0082(99)00064-7 [pii].

14. Tsai G, Goff DC, Chang RW, Flood J, Baer L, et al. (1998) Markers of glutamatergic neurotransmission and oxidative stress associated with tardive dyskinesia. Am J Psychiatry 155: 1207-1213.

15. Ozdemir V, Aklillu E, Mee S, Bertilsson L, Albers LJ, et al. (2006) Pharmacogenetics for off-patent antipsychotics: reframing the risk for tardive dyskinesia and access to essential medicines. Expert Opin Pharmacother 7: 119-133.

16. Hori H, Ohmori O, Matsumoto C, Shinkai T, Nakamura J (2003) NAD(P)H: quinone oxidoreductase (NQO1) gene polymorphism and schizophrenia. Psychiatry Res 118: 235-239. S0165178103000957 [pii].

17. Zai CC, Tiwari AK, Basile V, De Luca V, Muller DJ, et al. (2010) Oxidative stress in tardive dyskinesia: genetic association study and meta-analysis of NADPH quinine oxidoreductase 1 (NQO1) and Superoxide dismutase 2 (SOD2, MnSOD) genes. Prog Neuropsychopharmacol Biol Psychiatry 34: 50-56.

18. Liou YJ, Wang YC, Chen JY, Bai YM, Lin CC, et al. (2007) Association analysis of polymorphisms in the N-methyl-D-aspartate (NMDA) receptor subunit 2B (GRIN2B) gene and tardive dyskinesia in schizophrenia. Psychiatry Res 153: 271-275.

19. Ivanova SA, Loonen AJ, Pechlivanoglou P, Freidin MB, Al Hadithy AF, et al. (2012) NMDA receptor genotypes associated with the vulnerability to develop dyskinesia. Transl Psychiatry 2: e67. tp201166 [pii];10.1038/tp.2011.66 [doi].

20. Lee HJ, Kang SG (2011) Genetics of tardive dyskinesia. Int Rev Neurobiol 98: 231-264. B978-0-12-381328-2.00010-9 [pii];10.1016/B978-0-12-381328-2.00010-9 [doi].

21. Greenbaum L, Alkelai A, Zozulinsky P, Kohn Y, Lerer B (2011) Support for association of HSPG2 with tardive dyskinesia in Caucasian populations. Pharmacogenomics J . tpj201132 [pii];10.1038/tpj.2011.32 [doi].

22. Syu A, Ishiguro H, Inada T, Horiuchi Y, Tanaka S, et al. (2010) Association of the HSPG2 gene with neuroleptic-induced tardive dyskinesia. Neuropsychopharmacology 35: 1155-1164. npp2009220 [pii];10.1038/npp.2009.220 [doi].

23. Inada T, Koga M, Ishiguro H, Horiuchi Y, Syu A, et al. (2008) Pathway-based association analysis of genome-wide screening data suggest that genes associated with the gamma-aminobutyric acid receptor signaling pathway are involved in neuroleptic-induced, treatment-resistant tardive dyskinesia. Pharmacogenet Genomics 18: 317-323.

24. Reynolds GP (2004) Receptor mechanisms in the treatment of schizophrenia. J Psychopharmacol 18: 340-345.

25. Sachdev PS (2005) Neuroleptic-induced movement disorders: an overview. Psychiatr Clin North Am 28: 255-74, x.

26. Eichhammer P, Albus M, Borrmann-Hassenbach M, Schoeler A, Putzhammer A, et al. (2000) Association of dopamine D3-receptor gene variants with neuroleptic induced akathisia in schizophrenic patients: a generalization of Steen's study on DRD3 and tardive dyskinesia. Am J Med Genet 96: 187-91.

27. Kay SR, Fiszbein A, Opler LA (1987) The positive and negative syndrome scale (PANSS) for schizophrenia. Schizophr Bull 13: 261-276.

28. Xu Z, Taylor JA (2009) SNPinfo: integrating GWAS and candidate gene information into functional SNP selection for genetic association studies. Nucleic Acids Res 37: W600-W605. gkp290 [pii];10.1093/nar/gkp290 [doi].
